# Obesity moderates the complex relationships between inflammation, oxidative stress, sleep quality and depressive symptoms

**DOI:** 10.1186/s40608-018-0208-2

**Published:** 2018-12-03

**Authors:** Alanna V. Rigobon, Thirumagal Kanagasabai, Valerie H. Taylor

**Affiliations:** 1 Faculty of Medicine, University of Toronto, Toronto, Canada; 2 Department of Epidemiology, Biostatistics, and Occupational Health, McGill University, Montreal, Canada; 3 Women’s College Research Institute, University of Toronto, Toronto, Canada; 4 Women’s College Hospital, 76 Grenville Street Room E947, Toronto, ON M5S 1B2 Canada

**Keywords:** Body mass index (BMI), Obesity, Sleep, Inflammation, Oxidative stress, Depression

## Abstract

**Background:**

The relationship between obesity and depression is complex. This study assessed the impact of body mass index (BMI) on the link between BMI, inflammation, oxidative stress, sleep quality and self-reported depressive symptoms.

**Methods:**

We used data from the U.S. National Health and Nutritional Examination Survey 2005–2008 cycles (*n* = 9133; ≥20y). Depressive symptoms and sleep quality were determined from questionnaires. C-reactive Protein (CRP) was used as a biomarker of inflammation and γ-glutamyltransferase was used to assess oxidative stress. The relationship between depressive symptoms, sleep quality, and biomarkers were assessed with regression models. The moderating effects of BMI and sex were tested.

**Results:**

BMI was a significant moderator of the relationship between γ-glutamyltransferase and depressive symptoms (*p* = 0.02), but not CRP or sleep quality. Higher BMI increased odds of depressive symptoms in women (OR (95% CI): 3.92 (1.85–8.30) for BMI ≥25 to < 30 kg/m^2^; 3.17 (1.53–6.58) for BMI ≥30 to < 35 kg/m^2^; and 7.38 (2.11–25.76) for BMI ≥35 kg/m^2^). BMI was also a significant moderator of γ-glutamyltransferase levels in those with vs without depressive symptoms. Those with depressive symptoms had 24% poorer sleep quality compared to those without depressive symptoms after adjusting for inflammation, oxidative stress and other confounders.

**Conclusions:**

The link between oxidative stress and depressive symptoms may be particularly relevant for females and people living with obesity. People with depressive symptoms also have a substantial reduction in sleep quality. Thus, research should examine these relationships prospectively to inform and improve the mental health of the adult population in developed countries.

## Background

Evidence has established a bidirectional link between depression and obesity [1, 2]. Obesity is associated with an increased vulnerability for depression, secondary to factors such as chronic pain, reduced mobility and issues related to societal bias and stigma [1, 3, 4]. For example, obesity places increased workload on joints and increases pro-inflammatory cytokines that promote further joint destruction and arthritis, one of the most common causes of chronic pain and reduced mobility in adults [5–7]. Conversely, depression increases the likelihood of obesity, given that decreased activity, fatigue and appetite changes are core symptoms [1, 8]. Studies examining the link between these conditions also suggest a common inflammatory pathway [9, 10]. Patients with depression show elevated levels of pro-inflammatory cytokines, acute phase reactants such as C-reactive protein (CRP), chemokines, and cell adhesion molecules [11]. Concomitantly, treatment studies suggest that inhibiting the inflammatory cytokine tumour necrosis factor (TNF) may improve depressive symptoms in patients with high baseline inflammatory biomarkers [12, 13]. Obesity also has an inflammatory component, and much of its clinical pathology has been linked to obesity-associated chronic inflammation of white adipose tissue and the resultant increased circulating concentrations of inflammatory markers [12, 14]. Although a common inflammatory pathway has been hypothesized to explain some of the depression-obesity confluence, evidence of a biological link between obesity and depression is conflicting.

A second biological association between depression and obesity relates to oxidative stress, a condition defined as a persistent imbalance between antioxidant and pro-oxidant processes, where the end result is excessive production of reactive oxygen species (ROS) and reactive nitrogen species (RNS) [15]. Increased levels of oxidative stress biomarkers are observed in both obese and depressed states [16, 17]. Recent studies show that oxidative stress in combination with the pro-inflammatory mechanism plays an important role in the development of a number of psychiatric disorders [18, 19]. This has led to the hypothesis that the combination of these pathways forms the pathophysiological basis of depression.

Sleep quality is also thought to play a role in the proinflammatory state seen in depression and obesity. Both experimental and clinical studies show a bidirectional association between sleep and inflammation [20]. Sleep loss, for instance, is known to induce proinflammatory markers CRP, Interleukin-6 (IL-6) and TNF that are associated with depression and obesity [21–23]. Proinflammatory cytokines also influence sleep, and the administration of infectious agents can fragment sleep via cytokine expression [24, 25]. Restoration of sleep decreases the induction of inflammatory pathways, reduces symptoms of depression, and attenuates gains in fat mass [26, 27]. These findings suggest that sleep-related mechanisms are implicated in the immunologic pathways linking depression and obesity.

Indeed, multifaceted factors link the etiology of both obesity and depression. Our study aims to: (1) assess the moderating effect of obesity on the associations of depressive symptoms with inflammation (i.e. CRP), oxidative stress (i.e. γ-glutamyltransferase), and sleep quality; and, (2) determine which of these factors increase the odds of having depressive symptoms in a nationally representative sample of U.S. adults.

## Methods

### Participants

The U.S. National Health and Nutrition Examination Survey (NHANES) is a series of nationally representative cross-sectional studies designed to assess the health and nutritional status of the non-institutionalized civil population [28]. NHANES uses multi-stage, unequal probability, and cluster sampling methods to recruit its participants. Data is collected from personal interviews, standardized physical examinations, and laboratory samples by trained professionals. Ethical approval was provided by the National Center for Health Studies Ethics Review Board for the NHANES, and informed consent was obtained from all participants. Approximately 10,000 people are sampled bi-annually, and the initial sample of our analysis included 20,497 individuals from the NHANES 2005–2008 cycles. We excluded from our analysis individuals with missing sleep quality variables (*n* = 18) or depression data (*n* = 1371), pregnancy (*n* = 392), and individuals less than 20 years old, (< 20 y; *n* = 9583). We excluded children and adolescents from our analysis since their BMI categorization is based on percentile, and their information on certain behavioural measures (e.g., smoking and alcohol intake) are restricted. Our final analytic sample was 9133 participants.

### BMI

We used body mass index (BMI; kg/m^2^) to measure the degree of obesity. BMI was categorized as < 18.5, ≥18.5 to < 25, ≥25 to < 30, ≥30 to < 35, and ≥ 35 kg/m^2^ from the continuous BMI variable provided by NHANES. Standing height was measured as the maximum vertical size using a Stadiometer to the nearest mm. Weight was determined with a Toledo digital scale in pounds, which was then converted to kilograms with the automated system by NHANES.

### Sleep quality

Sleep habits of participants were obtained from 6 questions *[“In the past month, how often did you have trouble falling asleep?”, “In the past month, how often did you wake up during the night and had trouble getting back to sleep?”, “In the past month, how often did you wake up too early in the morning and were unable to get back to sleep?”, “In the past month, how often did you feel unrested during the day, no matter how many hours of sleep you have had?”, “In the past month, how often did you feel excessively or overly sleepy during the day?”,* and *“In the past month, how often did you not get enough sleep?”]* from the Sleep Disorders Questionnaire. Only 2005–2008 NHANES cycles’ questionnaires contain these questions, and thus, our analysis is restricted to these cycles. The Sleep Disorders Questionnaire was administered to participants aged ≥16, who reported their typical sleep habits on weekdays or workdays over the past month, and responses were coded as 0 = “Never”, 1 = “Rarely (1 time a month)”, 2 = “Sometimes (2–4 times a month)”, 3 = “Often (5–15 times a month)”, 4=“Almost always (16-30 times a month)”. Individual responses from the 6 questions were then summed to obtain a global sleep quality score for each participant [29] with a higher sleep quality score indicating lower sleep quality.

### Depressive symptoms and severity

The Patient Health Questionnaire (PHQ-9) was used to estimate prevalence and severity of depressive symptoms [30]. The presence of depressive symptoms was conferred based on participants’ responses to 9 depression-related symptoms over the last 2 weeks. Responses to each question were: “Not at all”, “Several days”, “More than half the days”, and “Nearly every day” (scored as 0, 1, 2, 3, respectively), which were summed and categorized: depressive symptom severity (“None”: 0–4; “Minimal”: 5–9; “Mild”: 10–14; “Moderate”: 15–19; and “Severe”: 20–27); Participants with none to mild symptom severity (i.e., score of 0–14) were categorized as having “no symptoms of depression”, and participants with moderate to severe depression severity (i.e., score of 15–27) were categorized as having “depressive symptoms” [31, 32].

### Inflammation and oxidative stress

Laboratory measures of CRP (nM) and γ-glutamyltransferase (U/L) were used to assess inflammation and oxidative stress, respectively [33, 34]. The average time between initial participant interview and medical examination center visit for laboratory collection was 2 weeks [35]. Description of the laboratory methodology, data processing, and quality assurance details for the biomarkers are available on NHANES’s website [36]. Briefly, CRP was measured in frozen serum using Nephelometry at the University of Washington Medical Center. Blood was collected using regular or serum-separator Vacutainers, and the serum was separated from the cells within 60 min of the collection. The samples were stored in tightly sealed vials to prevent desiccation and frozen at ≤20 °C until particle-enhanced assay could be performed. The assay uses latex particles coated with mouse monoclonal anti-CRP antibodies to quantify the presence of CRP in the samples as they form antigen-antibody complexes with the latex particles. γ-glutamyltransferase was measured in Refrigerated Serum using Beckman Synchron LX20 by Collaborative Laboratory Services, L.L.C. Separated samples were frozen at − 15 to − 20 °C and thawed until LX20 analysis. The LX20 uses an enzymatic method to quantify γ-glutamyltransferase activity at an absorbance of 410 nm. Participant fasting was not required for CRP or γ-glutamyltransferase sample collection.

### Covariates

Age, sex, ethnicity, education, income, smoking, alcohol intake, and recreational physical activity level were considered as potential confounders [37]. Age was also categorized as 20 to < 40, 40 to < 65, and ≥ 65 y. Ethnicity was self-reported and categorized as non-Hispanic White, non-Hispanic Black, Mexican American, and Other. Income was also self-reported and categorized as <$20,000, $20,000 to 44,999, and ≥ $45,000; and, education categorized as <high school, high school, and at least some college or above. Alcohol intake was categorized as < 3 or ≥ 3 drinks per day. Smoking was categorized as current (if smoking now), past (if they smoked ≥100 cigarettes in a lifetime) or never (if smoked < 100 cigarettes in a lifetime) [38]. Further, recreational physical activity level was calculated using the metabolic equivalent (MET) score assigned for each activity by NHANES and categorized as “inactive” (no reported recreational physical activity data), “somewhat active” (< 500 MET-min/week) and “active” (≥500 MET-min/week).

### Statistical analyses

Mean and 95% confidence interval (CI) for continuous variables, and percentage and 95% CI for categorical variables were determined according to presence or absence of depressive symptoms. Differences in demographic and behavioural characteristics of participants were assessed by independent t-test and χ^2^ analyses, as appropriate. PROC SURVEYLOGISTIC was used to assess the association of depressive symptoms (0, 1) with CRP, γ-glutamyltransferase, and sleep quality. PROC SURVEYREG was used to estimate CRP, γ-glutamyltransferase, and sleep quality in adults with vs without symptoms of depression. The moderating effect of BMI and sex on these associations were tested with interaction terms. Confounding variables adjusted in our models include age, sex, ethnicity, education, income, smoking, alcohol intake, and recreational physical activity. When appropriate, CRP, γ-glutamyltransferase, and sleep quality were also considered as confounders. All analyses are weighted to represent the U.S. adult population using SAS v9.4 (Cary, NC, U.S.A). Statistical significance was set at α < 0.05.

## Results

**Fig. 1:**
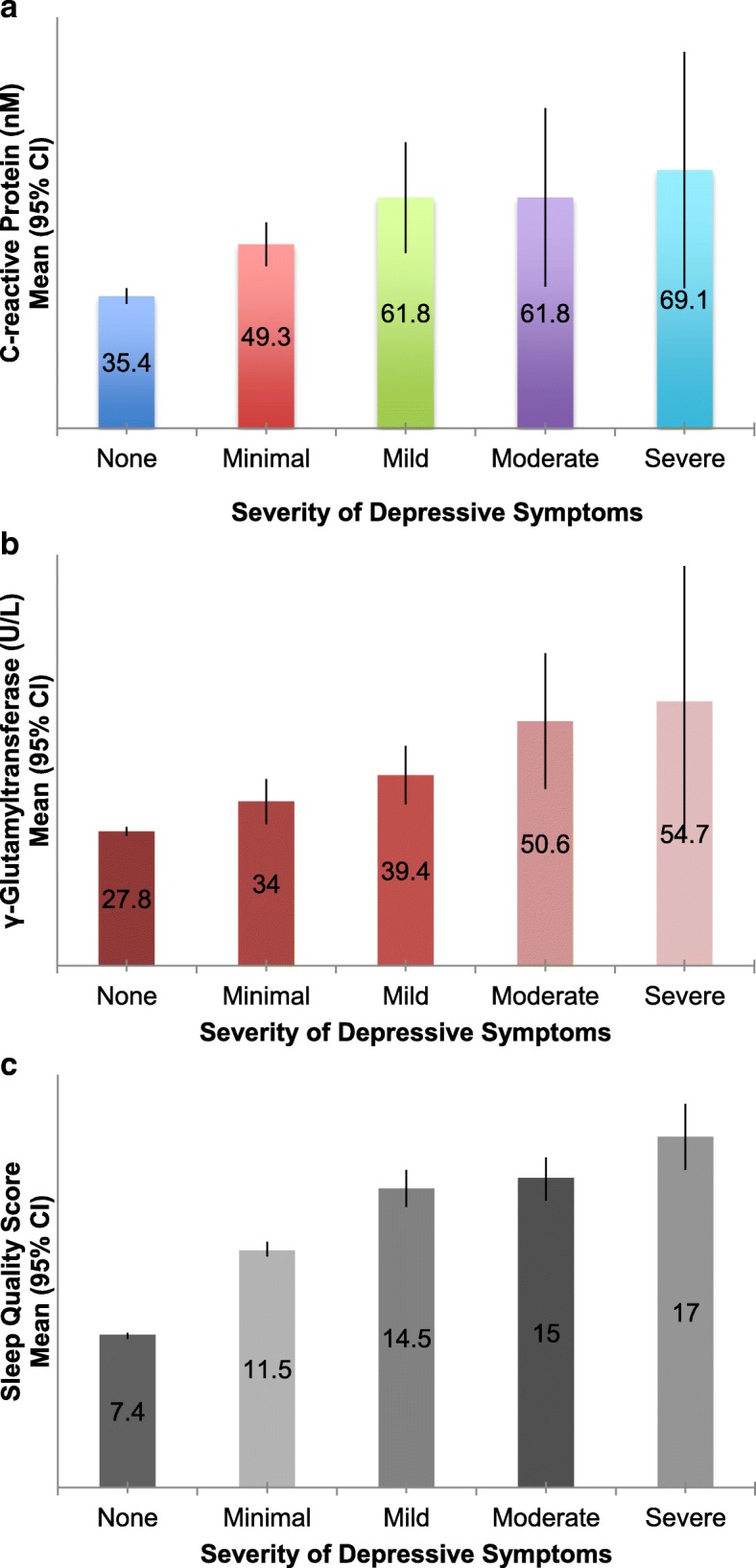
Higher severity of depressive symptom is associated with higher inflammation (**a**), oxidative stress (**b**), and lower sleep quality (**c**)

Table 1 describes the US adult population by the presence or absence of self-reported depressive symptoms. Those with depressive symptoms were more likely to be middle-aged (≥40 to < 65 y), women, Non-Hispanic Blacks, have lower education and income, and be current smokers. In general, higher severity of depressive symptoms was associated with higher inflammation and oxidative stress, and lower sleep quality (Fig. 1). Obesity was a significant moderator of the relationship between depressive symptoms and γ-glutamyltransferase (*p* = 0.02), but not CRP (*p* = 0.43) or sleep quality (*p* = 0.93). Rather, the later relationships were strongly confounded by sex (i.e., higher in women compared to men), income, smoking status, and physical activity (Table 2). The moderating effect of obesity on the former relationship between γ-glutamyltransferase and depressive symptoms was very weak (Table 3), and it may only be important in women. Indeed, of the covariates considered in our analysis, only being a woman was significantly associated with higher odds of having depressive symptoms and the magnitude of this association increased with BMI. Sex was not a moderator of the relationship between depressive symptoms and γ-glutamyltransferase (*p* = 0.31).

**Table 1:** Characteristics of the US adult population (≥20 y)

Characteristics	No Depressive Symptoms (*n* = 8888)	Depressive Symptoms (*n* = 245)	*P* value
Age (Mean (95% CI))	46.9 (46.1, 47.8)	46.6 (45.1, 48.1)	NS
Age categories (% (95% CI))			
≥ 20 to < 40 y	36.7 (34.8, 38.7)	31.1 (23, 39.2)	< 0.05
≥ 40 to < 65 y	46.0 (44.3, 47.7)	61.8 (52.1, 71.5)	
≥ 65 y	17.3 (15.5, 19.0)	7.1 (4.0, 10.2)	
Sex			
Men	49.6 (48.6, 50.6)	31.7 (25.2, 38.1)	< 0.05
Women	50.4 (49.4, 51.4)	68.3 (61.9, 74.8)	
Ethnicity			
Non-Hispanic White	72.1 (67.6, 76.6)	65.0 (55.9, 74.2)	< 0.05
Non-Hispanic Black	10.8 (8.1, 13.5)	17.3 (10.5, 24.2)	
Mexican American	7.9 (6.1, 9.8)	7.3 (3.7, 10.9)	
Others	9.2 (7.3, 11)	10.3 (5.1, 15.5)	
Education			
< High school	18.2 (16, 20.4)	29.5 (23.4, 35.5)	< 0.05
High school	25.0 (23.3, 26.7)	31.0 (21.6, 40.4)	
College	56.8 (53.4, 60.1)	39.5 (30.6, 48.4)	
Income			
< $20,000	15.5 (13.8, 17.3)	35.5 (30.5, 40.6)	< 0.05
$20,000-44,999	29.8 (27.6, 32.0)	38.8 (30.7, 47.0)	
≥ $45,000	54.7 (51.4, 57.9)	25.6 (18.4, 32.9)	
Smoking			
None	51.6 (49.4, 53.7)	38.4 (28.9, 47.9)	< 0.05
Current	23.2 (21.4, 25)	45.1 (35.9, 54.2)	
Past	25.2 (23.8, 26.6)	16.5 (11.2, 21.9)	
Alcohol Intake			
< 3 drinks per day	62.3 (60, 64.6)	56.1 (44.9, 67.3)	NS
≥ 3 drinks per day	37.7 (35.4, 40)	43.9 (32.7, 55.1)	
Recreational Physical Activity			
None reported	66.2 (62.6, 69.9)	84.4 (77.8, 91)	< 0.05
< 500 MET-min/week	11.4 (10.1, 12.7)	6.1 (2, 10.2)	
≥ 500 MET-min/week	22.3 (19.7, 24.9)	9.5 (5.4, 13.6)	
BMI categories (kg/m^2^)			
< 18.5	1.6 (1.2, 2.0)	1.1 (0.0, 2.2)	NS
≥ 18.5 - < 25	30.7 (29.0, 32.4)	24.3 (17.5, 31.2)	
≥ 25 - < 30	33.7 (32.5, 35.0)	31.5 (25.0, 38.0)	
≥ 30 - < 35	19.4 (18.5, 20.4)	21.9 (14.5, 29.3)	
≥ 35	14.5 (13.2, 15.9)	21.1 (14.2, 28.1)	

Mean (95% CI) for continuous variables and % (95% CI) for categorical variables. Responses from the PHQ-9 were summed and categorized as None (0–4), Minimal (5–9), Mild (10–14), Moderate (15–19), and Severe (20–27). Moderate-to-Severe was categorized as Depressive Symptoms. Sleep Quality are sum of responses for 6 sleep habit questions from the Sleep Disorder Questionnaire. *p* < 0.05, two-sided; t-test or Chi-square, as appropriate. *NS* not significant. Sum of weights = 190,972,201

**Table 2:** Multivariable model for C-reactive Protein and sleep quality and their association with depressive symptoms

Variable	OR (95% CI)	
	C-reactive Protein	Sleep Quality
C-reactive Protein	1.001 (1.001, 1.002)	–
Sleep Quality	–	1.190 (1.151, 1.231)
BMI	1.130 (0.958, 1.333)	1.108 (0.918, 1.339)
Age	0.999 (0.986, 1.012)	1.004 (0.989, 1.019)
Sex	2.336 (1.562, 3.492)	1.657 (1.098, 2.499)
Ethnicity	0.910 (0.726, 1.141)	0.967 (0.808, 1.158)
Education	0.974 (0.763, 1.242)	0.947 (0.732, 1.225)
Income	0.418 (0.332, 0.526)	0.472 (0.380, 0.587)
Smoking	0.789 (0.630, 0.989)	0.677 (0.515, 0.889)
Alcohol	1.415 (0.794, 2.520)	1.321 (0.739, 2.362)
Recreational Physical Activity	0.624 (0.470, 0.828)	0.658 (0.480, 0.903)

OR is the odds of having depressive symptoms adjusted for BMI, age, sex, ethnicity, education, income, smoking, alcohol, and recreational physical activity. All variables except for age were modeled categorically

**Table 3:** Multivariable model for the relationship between γ-glutamyltransferase and the odds of having depressive symptoms, stratified by BMI categories

Variable	OR (95% CI)			
	BMI categories (kg/m^2^)			
	≥18.5 - < 25	≥25 - < 30	≥30 - < 35	≥35
γ-glutamyltransferase	1.009 (1.002, 1.017)	1.004 (1.001, 1.007)	1.004 (0.999, 1.009)	1.003 (1.000, 1.005)
Age	0.996 (0.968, 1.025)	0.993 (0.972, 1.016)	0.971 (0.951, 0.991)	1.022 (0.989, 1.057)
Sex	1.218 (0.519, 2.858)	3.916 (1.848, 8.297)	3.168 (1.525, 6.580)	7.377 (2.113, 25.759)
Ethnicity	1.192 (0.783, 1.813)	0.586 (0.409, 0.839)	0.663 (0.419, 1.051)	1.213 (0.692, 2.125)
Education	0.963 (0.493, 1.879)	0.895 (0.520, 1.540)	0.795 (0.441, 1.433)	1.493 (0.913, 2.442)
Income	0.668 (0.390, 1.146)	0.387 (0.252, 0.593)	0.406 (0.222, 0.744)	0.256 (0.169, 0.389)
Smoking	0.854 (0.441, 1.654)	0.637 (0.410, 0.990)	0.882 (0.564, 1.379)	0.780 (0.438, 1.390)
Alcohol	2.242 (0.765, 6.569)	1.186 (0.394, 3.568)	0.955 (0.442, 2.066)	0.872 (0.335, 2.268)
Recreational Physical Activity	0.709 (0.421, 1.196)	0.709 (0.378, 1.332)	0.171 (0.018, 1.631)	0.607 (0.267, 1.377)

OR is the odds of having depressive symptoms adjusted for age, sex, ethnicity, education, income, smoking, alcohol, and recreational physical activity. All variables except for age were modeled categorically

Additionally, compared to participants without depressive symptoms, participants with symptoms had 24.65 nM (95% CI: 5.25, 44.06) higher CRP levels. This association also remained significant after adjustments for age, sex, ethnicity, education, income, smoking, alcohol, and recreational physical activity to 12.13 nM (1.43, 22.82) but became non-significant following adjustments for BMI (9.11 nM (− 0.31, 18.53)) and sleep quality (6.02 nM (− 3.45, 15.49)). BMI did not moderate of the association between CRP levels or sleep quality and depressive symptoms. The association between γ-glutamyltransferase and depressive symptoms was, however, moderated by BMI (*p* = 0.04). Compared to participants without depressive symptoms and BMI ≥18.5 to < 25, participants with depressive symptoms had significantly higher γ-glutamyltransferase levels: 2.41 U/L (0.88, 3.95) for BMI ≥25 to < 30; 8.67 (2.46, 14.87) for BMI ≥30 to < 25, and 12.35 (8.33, 16.37) for BMI ≥35, respectively) after adjusting for age, sex, ethnicity, education, income, smoking, alcohol, recreational physical activity and sleep quality. Finally, compared to participants without depressive symptoms, participants with depressive symptoms had significantly poorer sleep quality (i.e., the latter group had 5.84 (4.61, 7.06) higher sleep quality score out of a possible 24 points or 24.3% poorer sleep quality) after adjusting for sex, ethnicity, education, income, smoking, alcohol, recreational physical activity, CRP and γ-glutamyltransferase.

## Discussion

Results from this study are consistent with previous evidence that suggests inflammatory and oxidative stress are associated with depressive symptoms, obesity, and sleep disturbances [9, 20, 23, 39]. Indeed, we found that participants with self-reported depressive symptoms had higher inflammation and oxidative stress biomarkers, and poorer sleep quality compared to those without depressive symptoms. Our study also suggests that these relationships are lower than in some clinical studies—the lower effect sizes could be partly explained by the wider variations in both the exposure and outcome that are commonly found at the population level or the self-reported nature of depressive symptoms as an outcome. It has previously been hypothesized that the link between systemic inflammation, oxidative stress, and depression are associated with BMI [9, 23, 40]. We found a moderating effect for BMI on the relationship between y-glutamyltransferase and depressive symptoms, which augments current literature. The novelty of this study lies in our sex-specific findings, which suggest the link between oxidative stress and depressive symptoms may be particularly relevant for females. Female sex was a significant driver of this relationship, and higher BMI exacerbated the association between y-glutamyltransferase and depressive symptoms in women.

### Inflammation

The relationship between increased inflammatory biomarkers and depression has been well documented in the literature [23, 41]. In accordance with previous findings, our results showed slightly higher odds of depressive symptoms for inflammatory marker CRP. The lower magnitude of the association in our study could be due to multiple factors, including the self-reported nature of our outcome and the population level study design that is susceptible to higher variability. However, previous studies have implicated obesity as a possible moderating factor in this relationship [9, 23, 40]. Increased body mass appears to account for a portion of the relationship between depression and increased inflammatory markers [9, 23]. Consistent with this hypothesis, Miller and colleagues (2002) noted a synergistic relationship between depression and obesity with respect to CRP levels [40]. Indeed, depressive symptoms can facilitate weight gain over time as a result of sedentary behaviour [40, 42]. As fat cells accumulate, they produce leptin which acts to upregulate the expression of IL-6, thereby stimulating CRP, and promoting systemic inflammation [43]. Depression and fat mass are also thought to induce inflammation through their association with heightened Hypothalamic Pituitary Axis and Sympathetic Nervous System activity [44]. In particular, cytokines released in these inflammatory states can disrupt glucocorticoid receptor function, causing increased glucocorticoid resistance, and impaired negative regulation of Corticotropin-Releasing Hormone (CRH) by glucocorticoids [45]. Hyperactivity of CRH thereby leads to increased HPA and sympathetic response, further promoting inflammatory activation [11].

BMI did not moderate the relationship between inflammatory biomarkers and depressive symptoms in our study. However, we only tested one marker of inflammation (CRP) because other robust markers of inflammation (e.g., IL-6 and TNF-α) are not available in the NHANES data set, and the current utility of these inflammatory markers in depression is unclear [46]. CRP is a commonly used biomarker in the study of depression and obesity, and shows positive associations with both disease states [23, 47]. Further research is needed to examine the moderating and mediating effects of BMI on depression with additional biomarkers, e.g., malondialdehyde, F2-isoprostanes, 8-hydroxy 2′-deoxyguanosine, neuropeptide Y, adiponectin, IL-6, TNF-α, and IL-17.

### Oxidative stress

Previous studies exploring the relationship between oxidative stress and depression have used a variety of biomarkers to assess oxidative stress including F2 isoprostanes, 8-hydroxydeoxyguanosine and protein carbonyls [48]. The majority of these studies demonstrate an association between increased levels of oxidative stress markers and depression [17, 48]. The current study is the second to examine this relationship using y-glutamyltransferase and provides support for an association between depressive symptoms and increased oxidative stress for this biomarker [34], y-glutamyltransferase levels correlate with the body’s total antioxidant capacity and this biomarker has been used as a surrogate marker of oxidative stress in other NHANES-based analyses [34].

The depressed state is associated with compromised antioxidant responses, activated oxidative/nitrosative stress pathways and damage to fatty acids, DNA, and mitochondria [49]. It has been hypothesized that activation of oxidative stress pathways may increase susceptibility to neurodegeneration, and depression, as cells in the brain are at an increased vulnerability due to a high metabolic rate, low antioxidant levels, and an abundance of polyunsaturated fatty acids prone to oxidation [50, 51]. Our study was limited to the assessment of associations between these disease states, and further studies would be helpful to better delineate causality in order to understand underlying pathogenesis.

Oxidative stress pathways may be particularly relevant in individuals with obesity. Obesity is associated with hyperglycemia, increased muscle activity, elevated tissue lipid levels, inadequate antioxidant defenses, chronic inflammation, endothelial ROS production, and hyperleptinemia, which are all thought to contribute to oxidative stress [39]. Our results suggest a significant moderating effect of BMI on the relationship between y-glutamyltransferase and depression. Adipose tissue releases a host of immune factors that increase oxidative stress, and the accumulation of abdominal fat is specifically associated with elevations in lipid and protein peroxidation [52, 53]. It is possible that oxidative stress induced by fat tissue accumulation may interact or synergize with the increased oxidative response seen in depression, leading to differential effects in depressive patients according to BMI or adiposity classifications.

A novelty of our study lies with our sex-specific findings for the relationship between oxidative stress, obesity and depressive symptoms, which may be more relevant for females who show increased vulnerability to these disease states [54–56]. Clinical studies examining the effects of sex on oxidative stress in depressed patients are very limited and present controversial results [56–58]. A recent cross-sectional study in a small sample (*n* = 54) of healthy college students suggests the presence of a mild association between depressive symptoms and oxidative stress that is only present in females [58]. Females with depression also show increased levels of oxidative stress compared to non-depressed females [59]. Other researchers suggest that after sample stratification by sex, there is no association between oxidative stress parameters and clinical diagnosis of depression for females or males [60]. The reasons for inconsistency among these limited and early findings are not clear. It is possible that variation in the way depression is defined, i.e. self-reported accounts vs. clinical diagnoses, may affect the oxidative stress-sex relationship observed.

Results from our multivariable analysis corroborated findings by Matsushita and colleagues (2013) to suggest that female sex is a significant predictor of depressive symptoms for γ-glutamyltransferase [58]. Females showed increased odds of depressive symptoms and γ-glutamyltransferase across BMI categories and demonstrated a trend for increased odds of depressive symptoms and γ-glutamyltransferase with increasing BMI. Abdelkrim and colleagues (2015), suggest a variety of factors may contribute to these differences including fat tissue and distribution, hormonal differences, genetics, and variations in lifestyle [57]. Most relevant to our study is that an increased proportion of fat tissue in females may affect the oxidant/antioxidant balance. This is in line with research that suggests females have decreased antioxidant levels which are associated with adiposity/waist circumference [61].

### Sleep quality

Sleep quality represents an additional factor implicated in the inflammatory pathways that underlie depression. Studies from both healthy and depressed populations show an association between higher CRP levels and characteristics of sleep including disturbance, duration and quality [62–64]. Independent of inflammation, oxidative stress, and common covariates that affect the relationship between sleep quality and depression, our study is the first to report that people with depression have substantially reduced sleep quality. However, the definition of sleep quality remains unclear, and it may vary among various subpopulations. We also did not assess sleep latency, as this information was truncated at 60 min in both NHANES 2005–2005 and 2006–2008 datasets. Additional studies are needed to identify the appropriate dose-response relationship between sleep quality and inflammatory biomarkers in depression. These should also involve other data sets, which include objective measures of sleep quality such as sleep latency and efficiency.

### Strengths and limitations

Limitations of the study should be acknowledged. First, a cross-sectional design does not enable us to infer causality, and results must be interpreted cautiously. Additional longitudinal studies are needed to determine temporal sequences. Second, the relationship between obesity and depressive symptoms was examined using BMI, while indicators of central adiposity, including waist circumference, often demonstrate stronger associations with inflammation and oxidative stress [57, 61]. Third, we were unable to account for participants taking antidepressant medications, which may alter the levels of inflammatory and oxidative stress biomarkers [65, 66]. Fourth, depressive symptoms were based on self-reported questionnaires in the NHANES dataset and do not represent clinical diagnoses. All self-reported variables in our analysis are subject to recall and response biases. The majority of population-based studies examining the relationships between obesity and depression have also used questionnaire data to assess depression [1]. Fifth, there are an abundance of acute and chronic conditions, which may affect inflammatory markers studied, however it was not feasible to include all of these in the current study. Despite these limitations, our study provides novel evidence for a moderating effect of BMI on the relationship between oxidative stress and depressive symptoms in a large, nationally representative population. Further, NHANES provides sampling weights which take into account survey nonresponse, oversampling, post-stratification and sampling error to enable generalization of findings to the non-institutionalized civil adult population in the U.S.

## Conclusions

This study suggests that BMI may moderate the relationship between oxidative stress and depressive symptoms, particularly amongst females. We also found that people with depressive symptoms have substantially lower sleep quality independent of systemic inflammation and oxidative stress. Future work should be directed at examining this relationship longitudinally and with additional inflammatory and oxidative stress markers and alternative definitions of sleep quality to better understand the interactions among these pathways.

## Abbreviations

BMI: Body mass index
CI: Confidence interval
CRP: C-reactive protein
IL-6: Interleukin-6
NHANES: National health and nutrition examination survey
RNS: Reactive nitrogen species
ROS: Reactive oxygen species
TNF: Tumour necrosis factor
